# Medical Electricity

**Published:** 1904-06-04

**Authors:** 


### Progress in Medicine and Surgery.

### MEDICAL ELECTRICITY.

*(Concluded from page 156.)*

With a view to studying the actual rays of light to which the improvements clinically obtained in cases of lupus, etc., are to be attributed, Barnard and Morgan[8] have conducted certain experiments. These tend to show that the bactericidal rays exist mainly in the ultra-violet end, but those rays which are able to penetrate into the tissues and cause a reactionary effect are as yet not isolated. These authors have found that water diminishes the bactericidal power four-fifths, while this power is directly proportional to the intensity of the arc light used. The bacteria tested were B. coli communis, B. prodigiosus, B. subtilis micrococcus, tetragenus, staphylococcus aureus, and B. tuberculosis.

A new property of thorium has recently been discovered by Hugh Lieber[9] and substantiated by Tracy. When a fermentable substance is treated with the emanation from a radio-active substance such as thorium the rate at which it ferments is very greatly slowed. In this way grape juice has been kept in a warm room uncorked without fermenting for a month. Also the growth of a mould in a bottle containing agar-agar and starch after being exposed to the thorium emanations was greatly inhibited in comparison with that in a control bottle. Tracy suggests this property might be made of great use in medicine.

An attempt to bring fluorescence into immediate and intimate contact with the tissues has been attempted by W. J. Morton.[10] He urges that because a solution of quinine will fluoresce under the *x*-rays when in a strength of about 1 gr. to ℥viii. of water, the fluids of the body will also fluoresce if an appropriate dose of quinine be administered. This, he holds, should aid the radio-therapeutic results.

Marshall[11] finds an improvement upon Einhorn's method of using electricity for the treatment of gastric conditions in introducing a smaller deglutable electrode, and using for external stimulation two sponges connected by means of a V-shaped cord. One sponge is applied to the pneumogastric nerve in the neck on alternate sides, and the other, the larger, over the gastric area. He finds that with this method there is greater motor stimulation, but has not yet had opportunity to test the improvement which should be obtained in gastralgia.

W. J. Morton's[12] interesting record of cases comprises a surprising result of the *x*-rays upon a large carbuncle on the back of the neck. With a short application of the rays resolution took place, which three days later was increased by a second dose. The carbuncle burst on the fifth day, but only cheesy matter was extruded with a "core" 2½ inches long. The wound was healed in 11 days from commencing treatment, and the patient was so free from pain during the time that he had attended to his usual occupation in court and office the whole while. Two cases of keloid were relieved, though at the expense of a running reaction. A case of a fibroid tumour of the uterus with menorrhagia and pressure pains was treated with a high tube for 20–30 minutes a sitting. After 39 treatments the menstruation had become normal, the tumour had decreased in size considerably, and the patient was able to get about comfortably.

Having noted by accident that static electricity had improved the hearing of a patient who was being treated for other troubles, Fergusson[13] has treated four other cases of deafness with the high-frequency currents, and improvement has ensued in all. In one case a complete cure is reported—this being the only case in which sufficient time had been allowed for treatment. The causes of the deafness are not stated. One was an affection of the middle ear, another had vertigo, but it is not stated whether this was improved. The method used was the bipolar effleuve with brush electrodes held opposite both ears without sparking for five minutes and 10 minutes in the large auto-conduction solenoid. Some of the patients had been deaf for a long time.

Another case of mycosis fungoides has yielded satisfactory results in the hands of Carrier,[14] who treated it by means of *x*-rays. The result can be summed up in a few words as follows:—The treatment lasted from August 17th to November 10th, and at the end there was complete loss of induration, scaling, erythema, tumours or pruritus. The tube used with most effect was a high one, giving a 5-inch spark used for five minutes daily.

Sailer and Pfahler[15] draw attention to a very important point in the diagnosis of aneurysm of the aorta. In 18 cases which showed a pulsative swelling to the left of the sternum on the fluorescent screen, there were varying symptoms including in some cases tracheal tug and inequality of the radial pulses, while murmur, thrill, suprasternal pulsation, inequality of pupils, accentuation of the aortic second sound were also present in some. In four of the 18 cases autopsy revealed no aneurysm of the arch but an atheromatous and tortuous condition of the aorta. This then will rather tend to militate against Sansome's statement that the symptoms of an atheromatous condition are pain, dyspnœa, and occasional anginal attacks, while apparently there is no inequality of the pupils or pulses, no tracheal tugging or suprasternal pulsation. Dilatation gives rise to a shadow seen on either side of the sterum, and is readily confused with aneurysm, the differential diagnosis being made from the fact that there are no pressure symptoms, or tendency for the shadow to enlarge. In simple dislocation of the aorta the pulsating shadow may extend 6 inches to the left of the left border of the sterum, *i.e.* further than aneurysm of the arch would extend. In cases where the heart is elevated the natural bending of the aorta to the left is increased. In 46 cases that Holzknecht observed in which this shadow was noticed only six proved ultimately to be aneurysm.

[8] Brit. Med. Jour., Nov. 14. [9] Med. Rec., Jan. 23, 1904. [10] Ibid., Aug. 8, 1903. [11] Ibid., Aug. 8, 1904. [12] Ibid., July 25, 1903. [13] Brit. Med. Jour., Oct. 24. [14] Ibid., April 9, 1904. [15] Amer. Jour. Med. Sci., Oct. 1903.

